# On immature and adult forms of *Trichognathus marginipennis* Latreille, 1829 (Coleoptera, Carabidae, Galeritini)

**DOI:** 10.3897/zookeys.212.2705

**Published:** 2012-07-30

**Authors:** Guilherme Ide Marques dos Santos

**Affiliations:** 1 Museu de Zoologia da Universidade de São Paulo, Av. Nazaré, 481, Ipiranga, 04263-000, São Paulo, SP, Brazil

**Keywords:** *Trichognathus*, Galeritini, biology, Brazil, immatures, taxonomy

## Abstract

The pupa of *Trichognathus marginipennis* Latreille, 1829 is described for the first time and the adult is redescribed. Habitus and important structures of larva, pupa and adult are illustrated. This work improves the knowledge on immatures and adults of *Trichognathus marginipennis*.

## Introduction

*Trichognathus marginipennis* Latreille, 1829 is the only species in the monobasic genus. Reichardt (1964) considered *Trichognathus cinctus* Chaudoir, 1848 and *Trichognathus immarginipennis* Steinheil, 1875 as variants of *Trichognathus marginipennis* and synonymized the three names. A Neotropical precinctive, the known geographical range of this species includes the South American countries of Venezuela, Brazil, Colombia, Peru, Bolivia, Paraguay and Argentina, with all localities being east of, or on the lower eastern slopes of, the Andes mountain range.

Reichardt (1967), in his magisterial taxonomic monograph of the American Galeritini redescribed the adult stages of *Trichognathus* Latreille and its single species, *Trichognathus marginipennis*. Ball (1985), published a study of the galeritine genera, including *Trichognathus*, featuring characteristics of mandibles and ovipositors, two character systems that Reichardt had not studied. These features and others were illustrated with partially labeled scanning electron microscope images, *Trichognathus marginipennis* was again redescribed in a study about carabids of Colombia (Martinez 2005). Hunting (2008) also included this species in his study of the internal female genital system of galeritines.

The knowledge of immatures of Galeritini is very incipient. According to Arndt & Drechsel (1998), the few studies conducted on larval and pupal stages were related to *Galerita* species: *Galerita lecontei* Dejean, 1831 by Sallé (1849); *Galerita nigra* Chevrolat, 1861 and *Galerita simplex* Chaudoir, 1852 by Candèze (1861); *Galerita janus* (Fabricius, 1792) by Schaupp (1882); a redescription of *Galerita janus* by Kirk (1980); and the description of the last instar larvae of *Galerita brasiliensis* Dejean, 1826 and *Galerita carbonaria* Mannerheim, 1837 by Costa et al. (1988). Some notes about larval characteristics of Galeritini were made by van Emden (1942) and others by Thompson (1979) based on *Galerita lecontei* and *Galerita bicolor* Drury, 1773. Arndt and Drechsel described in 1998 the third instar larva of *Trichognathus marginipennis* and compared it with *Galerita* larvae in general.

In relation to the pupal stage, only that of *Galerita carbonaria* was described by Costa et al. (1988).

In the present study, the pupa of *Trichognathus marginipennis* is described for the first time and is compared with that of *Galerita carbonaria*. The adults of *Trichognathus margini-pennis* are redescribed and details of external structures, including metathoracic wing venation, are illustrated, as well as male and female genitalia. The larva is compared with a previous description (Arndt and Drechsel 1998) and the differences noted.

## Material and methods

Dozens of *Trichognathus marginipennis* (adults and larvae) were collected on July 31, 2008, on the sandy banks of the Rio Verde at Fazenda Soledad (Fig. 9c) in Campo Novo do Parecis, Mato Grosso, Brazil, after sunset.

The larvae and adults were transported to the lab in single plastic capped pots with some sand as substrate. The larvae were kept in the laboratory to obtain the pupae and the adults. Reared adults confirmed that the larvae and adults collected together are conspecific.

At the laboratory, each larva was transferred to a plastic covered pot approximately 8cm in diameter and 12cm in height containing medium-grained sand as substrate. Larvae of *Tenebrio* sp. (Tenebrionidae) were used as food, but as they were very large it was necessary to cut them into pieces and these were offered on tweezers to the carabid larvae. The adults were transferred to a large glass bowl (15cm in diameter and 9cm in height) with the same substrate used for the larvae; a large Petri dish (16cm diameter) was used as cover.

In order to fix the pupae, they were quickly boiled in water to extend them and to prevent the appearance of roughness when transferred to alcohol. Five larvae, two pupae and six adults were fixed and deposited in MZSP (Museu de Zoologia da Universidade de São Paulo) collection. All the examined material is deposited in MZSP.

For illustrations, the pupa was placed in a transparent container with alcohol gel. The alcohol gel is more viscous and prevents the specimen from changing position. A stereomicroscope with drawing tube was used. The pencil sketches were scanned and digitally redesigned with Adobe Photoshop CS4 and Adobe Illustrator CS4.

The same process for the illustration of pupa was used to prepare the larvae.

The living animals were photographed with a Canon Rebel XT DSLR camera with Canon EF 100mm 2.8 Macro lenses; a Canon Speedlite 430EX was used, too. Some reflectors made of paper were placed beside the specimen to improve the lighting.

The photo of the lateral view of the adult´s pronotum and head, as well as external views of the female genitalia, were made from pinned specimens of the MZSP collection using a Leica M205C stereomicroscope with a Leica DFC295 camera coupled. The photos of the different layers were combined with the Helicon Focus software or Leica Application Suite (Version 3.4.1). The same equipment and process was used to photograph the larva. The detail of the mandible was photographed with a Nikon Coolpix 4500 digital camera on the ocular of a Zeiss Axioscope 20 microscope.

Terms are based on Reichardt (1977) for general morphology, Snodgrass (1935) and Ball et al. (2011) for the mouthparts, Kukalová-Peck and Lawrence (1993) for the metathoracic wing, Deuve (1993) for the male genitalia, and Hunting (2008) for female genitalia.

The *Galerita carbonara* immature characters were based on the description and drawings by Costa et. al (1988).

## Descriptions

### Trichognathus marginipennis

Latreille, 1829

http://species-id.net/wiki/Trichognathus_marginipennis

#### Pupa.

**Figure 1.**
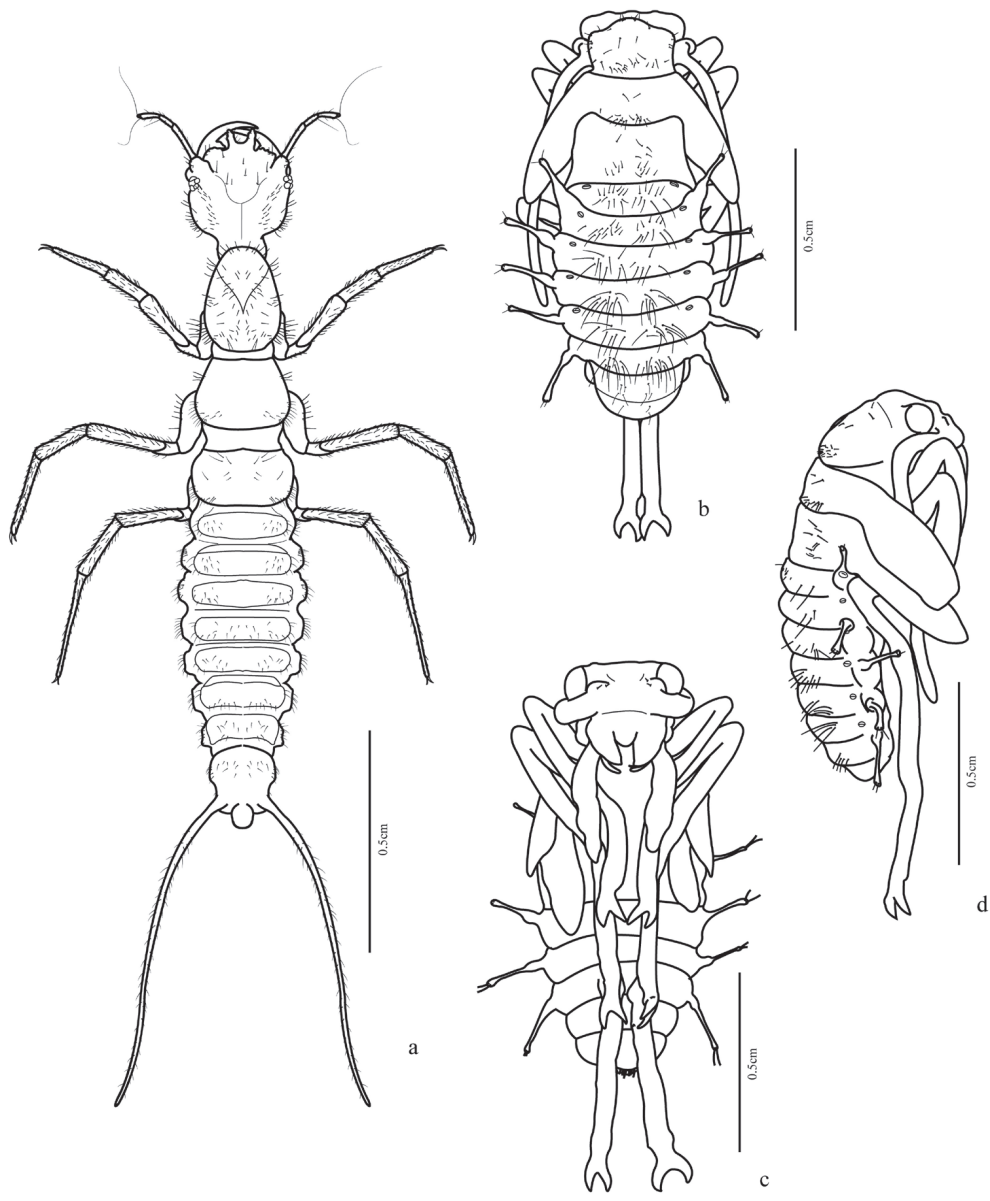
*Trichognathus marginipennis*: **a** habitus of third instar larva in dorsal view; habitus of pupa **b** dorsal view **c** ventral view **d** lateral view.

(Figs 1b–d, 7e–g) Body length: 11.0–12.0 mm.

Adecticous, exarate. Yellowish white with legs long and thin, antenna with two setae at base of scape. Antenna long, longer than half body length, folded posteriorly and fitted at inferior margin of eye, directed through ventral surface of legs. Eyes prominent, each about ¼ width of head. Labrum with rounded anterior margin. Mandible relatively long and markedly sclerotized at apex. Pronotum subtrapezoidal, anterior margin prominent and rounded at middle; many widely spaced setae, these more concentrated near margins. Mesonotum wider than pronotum with setae at middle, these more concentrated near base. Metanotum longer than mesonotum; higher concentration of setae on median region; metathoracic legs longer than abdomen. Abdomen with eight segments visible in dorsal view. Five pairs of lateral projections, wide at base, extended over a narrow stalk, widened slightly at apex, with a pair of short setae. The first lateral projection at junction of first and second visible abdominal segments, second from third segment, third from fourth and so on until sixth segment. Terga one to six dorsally with a marked concentration of long setae, these formed in two groups close to the midline. Segments seven and eight with many fewer setae. Spiracles visible in segments one to six, above the base of the lateral projections.

#### Adults 

(Figs 2a–f, 3a–j, 4a–e, 5a–h, 6a–d, 7b–d, 8a, 8f, 9a). Body length**:** 16**–**18.5mm.

Body form: dorso-ventrally depressed.

Color: head and pronotum from yellowish-brown to dark-brown; elytra dark-greenish with lateral margins and apex yellowish (Fig. 7b), legs from light to dark**-**brown, femora the same color as pronotum, some specimens with distal third darker;

Vestiture: integument covered dorsally and ventrally with testaceous setae; those of antennae and especially the scape, noticeably longer and more robust than on the other segments.

**Figure 2.**
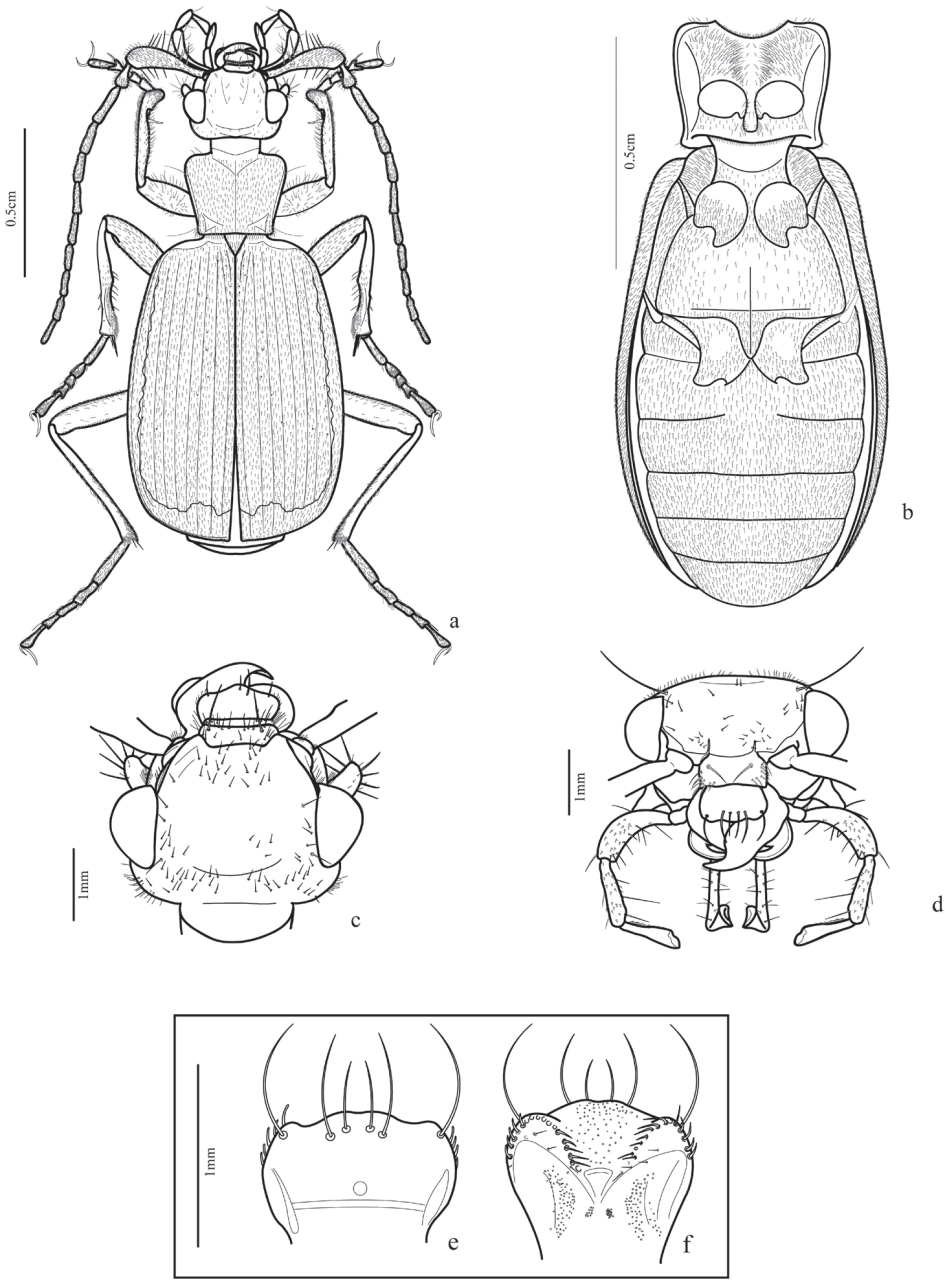
*Trichognathus marginipennis* (adult male): **a** habitus dorsal **b** thorax and abdomen in ventral view **c** head in dorsal view **d** head in front view; **e**. labrum, dorsal view **f** epipharynx, ventral view.

Head (Figs 2c–d, 7d): wider behind eyes; two pairs of long orbital setae, short scattered dorsal setae, sparser at middle ventrally, suborbital zone with an elevation near the inner margin bearing 5-10 thick setae, like those of scape and some sparser and shorter. Gula glabrous.

Antenna (Fig. 2a): long (approximately ^2^/_3_ of the body length) and covered with short setae; antennomere 1 very long, as long as antennomeres 3 and 4 together, robust, club-shaped bearing long ventral thick setae; antennomere 2 short widened at apex**,** with thick ventral setae, antennomeres 3-11 setiform.

Labrum (Figs 2e–f): subrectangular, lateral margins rounded, lobed anteriorly; six long apical setae: one on each angle and four near middle. Epipharynx with two rounded rows of anterior parapedial setae converged medially, each row bearing approximately 13 setae; median region with thin setae near the thick ones.

**Figure 3.**
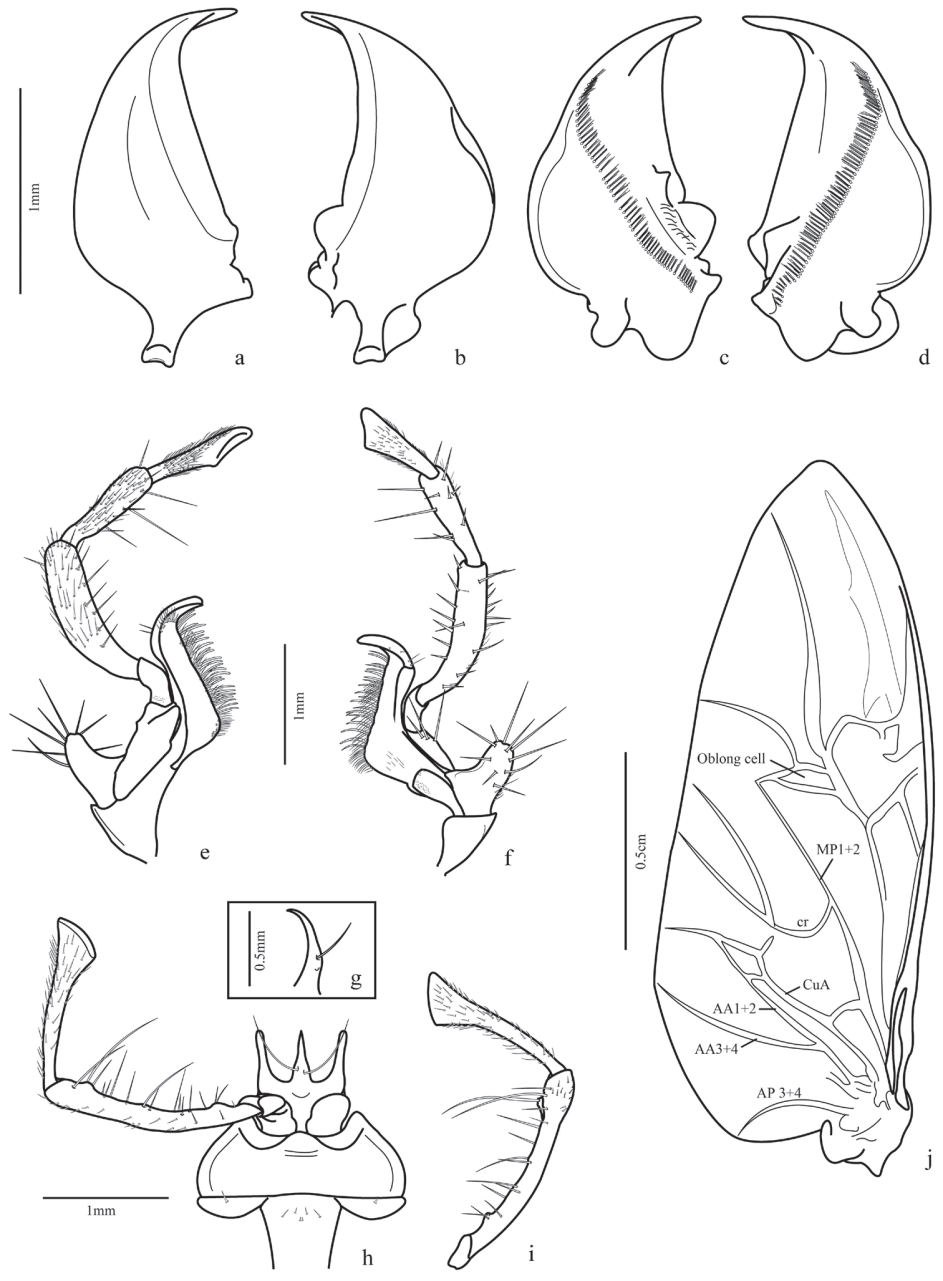
*Trichognathus marginipennis*, male: **a** left mandible in dorsal view **b** right mandible in dorsal view **c** right mandible in ventral view **d** left mandible in ventral view; maxilla **e** dorsal view; f. ventral view **g** glossa in lateral view **h** labium in ventral view **i** labial palp in dorsal view **j** hind wing – **AP**: anal posterior **AA** anal anterior **CuA** cubitus anterior **MP** media posterior.

Mandibles asymmetrical (Figs 3a–d): incisor region 3-4 times longer than molar region, terebral margin smooth, scrobe glabrous, anterior retinacular tooth prolonged ventrally; ventral groove (fig. 8f) crosses entire mandible with many ventral microtrichia inserted in each puncture.

Maxilla (Figs 3e–f): basistipes with lobular projection and about ten spine-shaped setae and a few thin setae; lacinia with tooth-shaped apex with setae concentrated on inner margin and a row of thicker setae, basal pubescence present; galea two segmented, basigalea longer than distigalea with some setae; palpifer with three thick spine-shaped setae; palpomere 1 with one seta and many punctures near the base, palpomeres 2 and 3 with fine pubescence and ventral spine-like setae; palpomere 4 securiform and covered with fine pubescence.

Labium (Figs 3g–i): submentum-mentum suture present; mentum transverse, emarginate, its lateral lobes with acute apices and rounded tooth between them, palpiger glabrous, longer than palpomere 1, palpomere 2 longer than 3, covered with setae and some long ventral ones; palpomere 3 securiform and covered with fine pubescence; prementum with each paraglossa narrow, separated from glossal sclerite, latter unciform, upwardly directed (Fig. 3g) with two long setae ventrally, on proximal third.

Prothorax (Figs 2a–b, 7d): subtrapezoidal, height in lateral view similar to width in dorsal view (pronotum slightly convex and prosternum markedly convex), anterior margin slightly larger than posterior one, anterior angles more widely rounded than posterior ones; pronotum covered with fine setae, one or two longer setae on the anterior third of the margins and another one next to posterior angles. Proepisternum with short setae near anterior margin. Prosternum covered with many thick setae and a few thin ones, high concentration of thick setae near the midline. Proepimeron dark with few sparse thin setae.

Mesothorax (Fig. 2b): with mesosternum darker laterally, covered with setae; mesepisternum subrectangular, dark, with margin adjacent to mesocoxae wider than its opposite and covered with thin setae; mesepimerum dark and usually glabrous.

Metathorax (Fig. 2b): metasternum wide, darker laterally, pubescence more concentrated laterally and near posterior margin; metepimeron elongate, dark and glabrous with blunt and wide proximal portion that partially covers ventrite I, middle region more tapered and wide inner margin.

**Figure 4.**
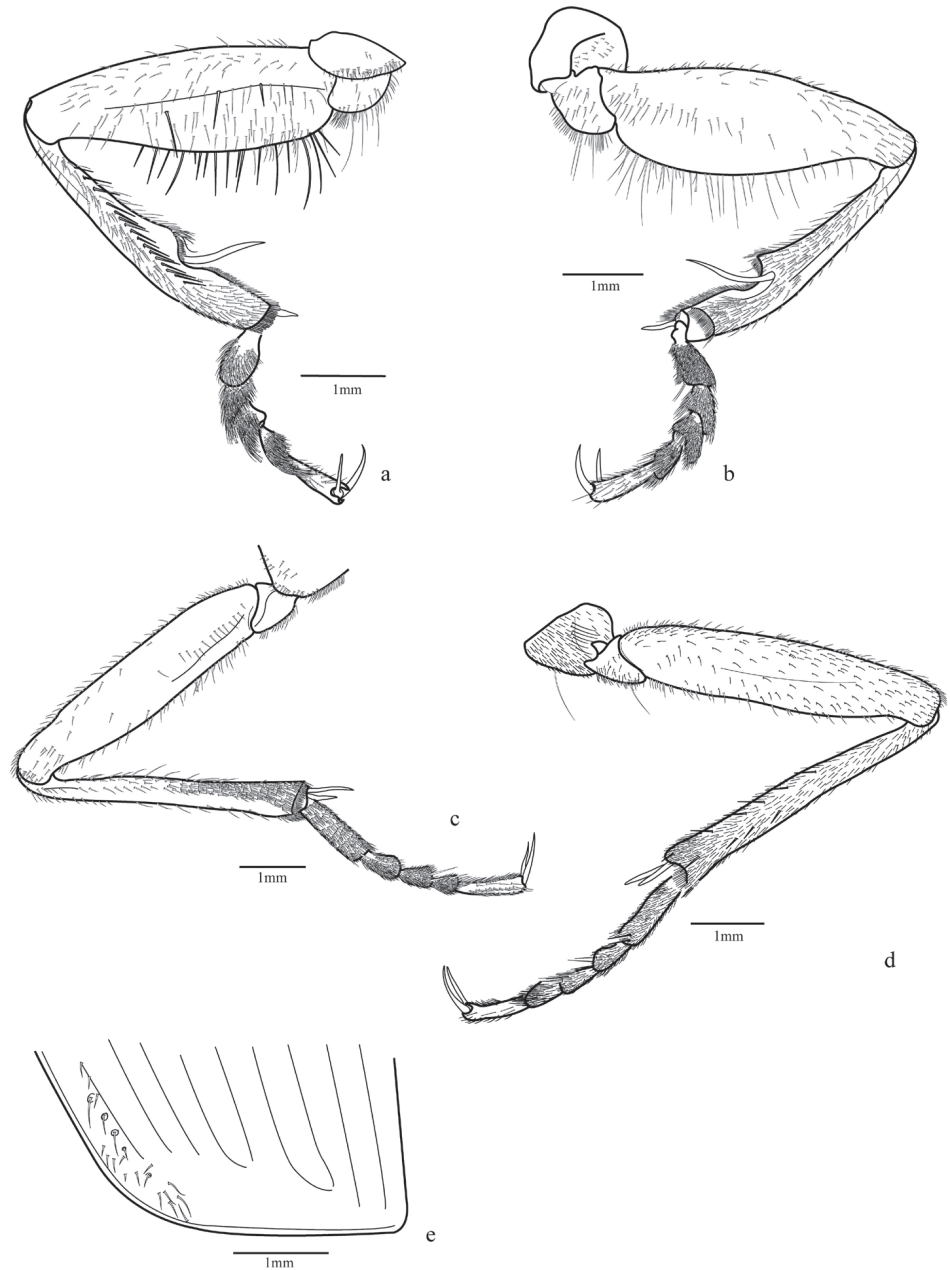
*Trichognathus marginipennis*, male. Fore leg **a** external view **b** internal view; median leg **c** internal view **d** external view **e** elytra apex.

**Figure 5.**
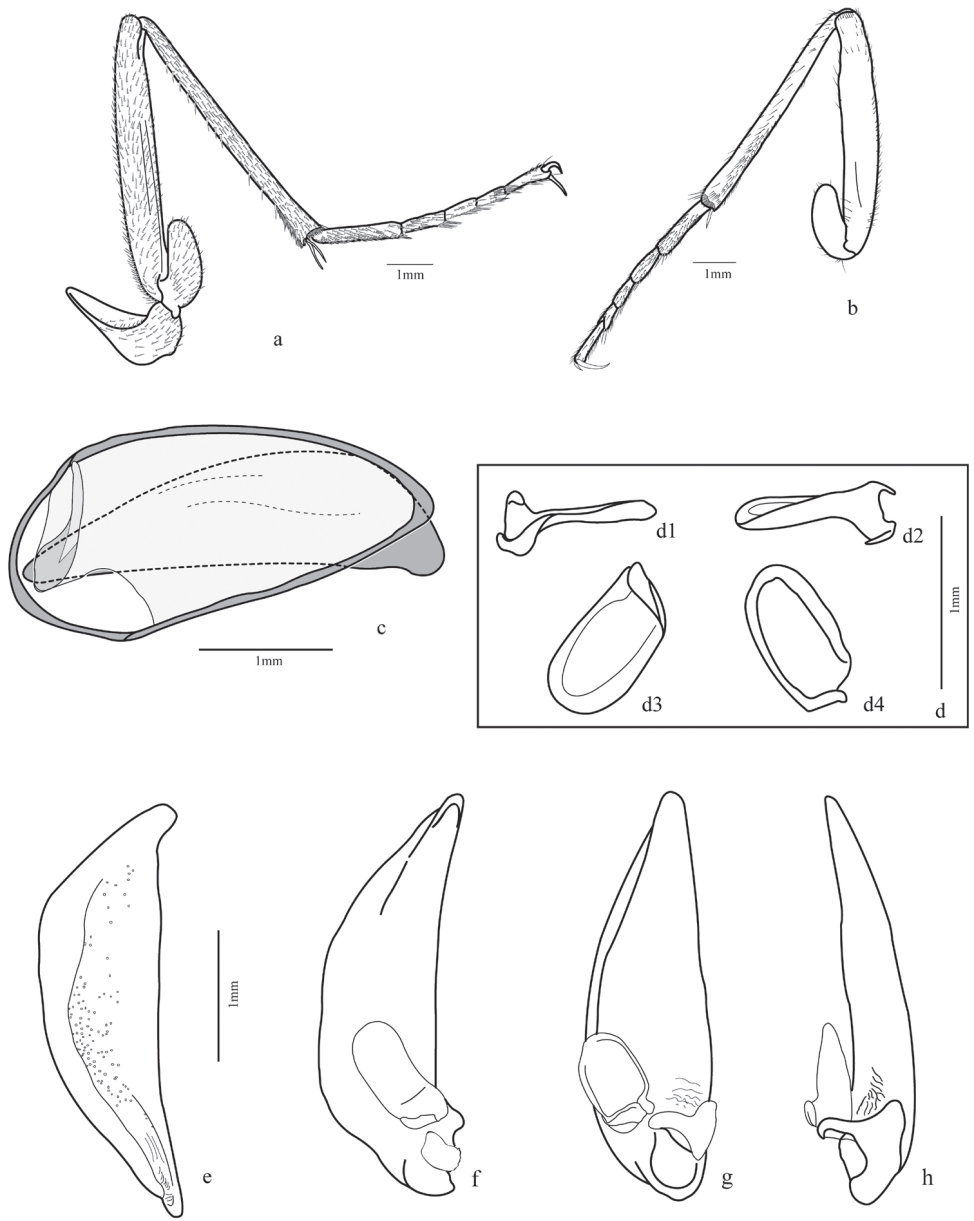
*Trichognathus marginipennis*, male. Hind leg: **a** external view **b** internal view **c** male genital segments (median lobe in dash line) **d** left paramere in lateral view (**d1** and **d2** lateral views **d3** ventral view **d4** dorsal view) **e** median lobe in side view; aedeagus **f** lateral view **g** ventral view **h** ventro-lateral view.

Legs (Figs 4a–d; 5a–b; 9b): fore and median coxae globose covered with thin setae, hind coxa with only few setae at middle and one or two long setae near anterior margin; median coxa with one or two long setae near the posterior margin; trochanter subtriangular, fore trochanter with a few sparse short setae on entire surface and thick and long ones on ventral margin; median trochanter with one or two long setae ventrally; femur long and covered ventrally with short setae, fore femur broader than others, flattened laterally with outer surface with a band of thicker and longer setae midline, inner surface with more concentration of setae near base, ventral margin with very long and thick spine-like bristles**;** hind femur longer than others; tibial length subequal to femur, tibia covered with thin setae and two dark spurs at the apex, except the fore tibia with widely separated spurs, only one distally; antenna cleaner on the distal third with a strong dark spur curved at base and a row of thick setae on the inner surface extended from base to apex; median and hind tibiae with more concentrated pubescence ventrally, especially on posterior half and two dark spurs at apex; tarsomeres 1-4 covered with setae, denser ventrally; fore tarsomeres 1-3 wider in males with rows of adhesive setae with a wide and rough plate (Fig. 9b); tarsomere 5 elongate with only few setae; tarsal claws simple and glabrous.

Elytra (Figs 2a, 4e): stria 1 very short, ranging from level of scutellum to approximately anterior fifth terminated in thick punctuation; fine setae covering entire structure with a few longer bristles in more defined punctations, especially near stria 1; posterior third near external margin and in the apex; striae 4 and 5 very close to one another at the apex, as are striae 6 and 7.

Hind wing (Fig. 3j): fully developed, about 2.5 times longer than wide, rounded anal lobe and tapered apex; anal vein bifurcated near base, proximal branch reaching margin and other branch (AA1+2) fused with cubitus anterior (CuA) at level of posterior third, forming a cell; a small branch extended from cell formed to cr branch, but not connected; oblong cell present.

Male genital segments (Fig. 5c): tergite IX formed by antecostal region, a tergite and two laterotergites; antecostal region arc shaped, mediotergite IX shorter and less curved; laterotergites IX form a comma-shaped structure.

Aedeagus (Figs 5d–h): median lobe cylindrical, slightly curved downward; phallobase rounded and distal region tapered with very short tip, dorsal surface slightly sclerotized and wrinkled with many punctures except anterior fifth and at the base; left paramere (Fig. 5d) broad, concave, base deflected and left basal projection going over the base; right paramere smaller and markedly attached at base of median lobe.

**Figure 6.**
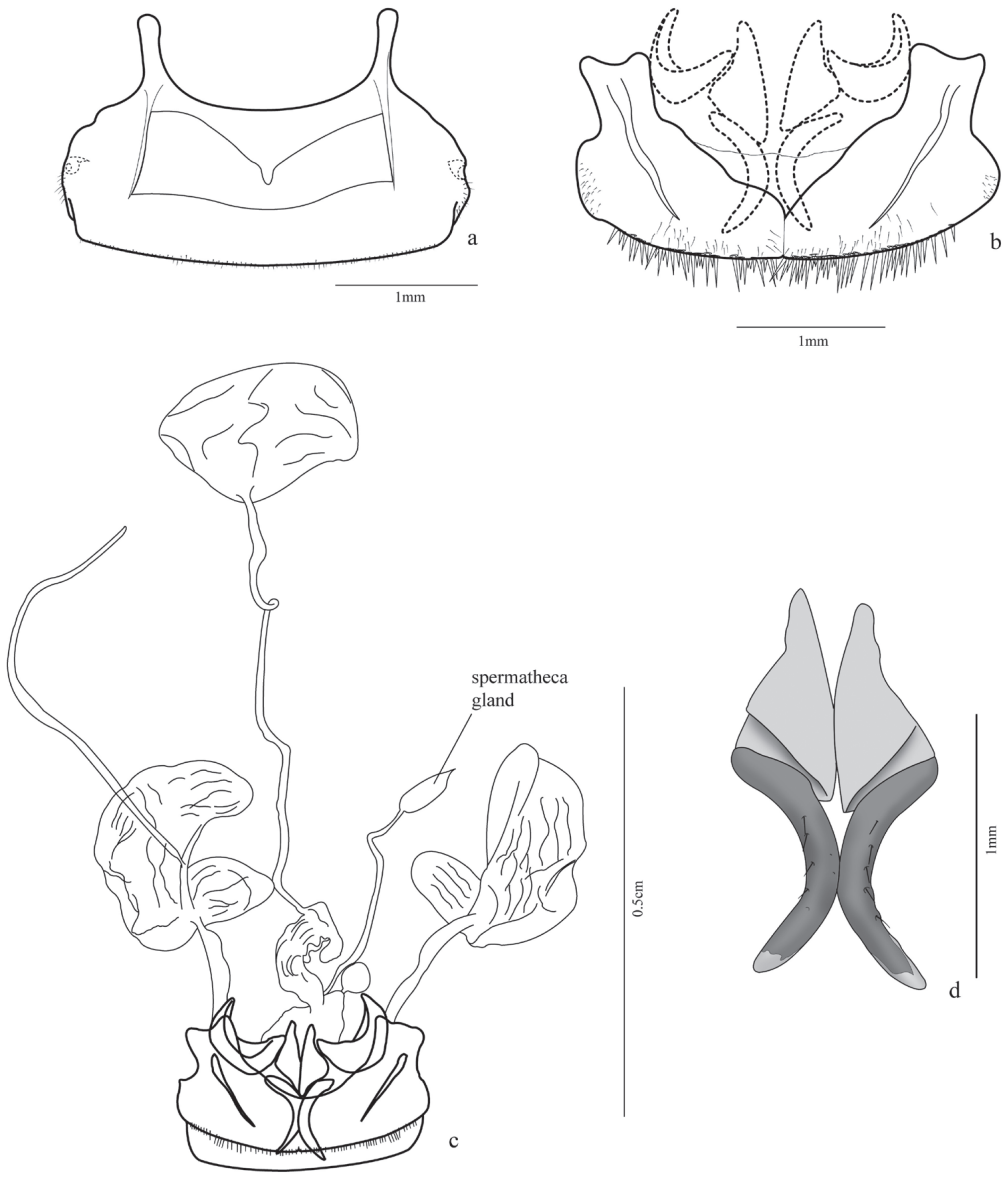
*Trichognathus marginipennis*, female **a** sternite VIII **b** tergites VIII (laterotergites IX, gonocoxites 1 and 2 in dash line) **c** female genitalia and pygidial glands **d** gonocoxites 2.

Female genital segments: sternite VIII (Figs 6a, 9a) 2.45 times wider than long, sclerotized, more weakly at medio-basal region; two basal projections (apodemes) almost half size of ventrite length, with rounded apex; fine setae posterior to spiracles and bordering posterior margin; two transverse lateromedian sclerotized stripes from base of projections to distal third of ventrite. Tergum 8 (Fig. 6b) formed by two laterotergites united at middle by a thin membrane, each laterotergite with a wide projection at the anterior margin, slightly bilobed at apex, and a lateral sclerotized band similar to that of sternite VIII, short thin setae at lateroexternal margin and distal margin with many long spine-like setae and a few thinner ones.

Female genitalia (Figs 6c–d, 8a). Ovipositor with gonocoxites 1 subtriangular, falciform; gonocoxites 2 with apex turned out and some setae near inner margin, laterotergite IX folded upon itself in internal margin, thus half-moon shaped. Internal organs comprised of a wrinkled bursa coiled at base; secondary spermathecal gland globose; spermathecal gland fusiform, attached by long duct, spermatheca 2 of similar size to pygidial glands, but more rounded.

#### Examined material.

BRAZIL, Amazonas: Rio Juruá, 1902, E. Garbe col., Departamento de Zoologia São Paulo, 1♀, 2♂. Mato Grosso: Barra do Garças, IX.1943, Departamento de Zoologia São Paulo, 2♀; Campo Novo do Parecis, 25.VII – 04. VIII, S. Rosa, S. Casari, G. Ide & L. Prado cols., 4♀, 2♂; Corumbá, XI.1917, E. Garbe col., Departamento de Zoologia São Paulo, 1♀, 1♂ ; Diauarum, 02.XI.-11.XII.1973, G. R. Kloss col., 4♀, 5♂; Três Lagoas, Fazenda Floresta, 13-20.IX.1964, Exp. Depto. Zool., 6♀, 1♂; same locality, Fazenda Retiro de Telhas, 16.X.1964, Exp. Depto. Zool. col., 1♀; same locality, left margin Sucuriu river, Fazenda Canaã, XI.1966, F. Lane col., 2♂; same locality, X.1966, F. Lane col., 13♀, 7♂ ; same locality, IV.1967, F. Lane col., 1♀, 1♂; same locality, I.1967, F. Lane col., 8♀, 5♂; same locality, VI.1967, F. Lane col., 2♀, 3♂. Minas Gerais: Paracatu, VII. 1960, Exp. Formoso col., 1♀. São Paulo: Boa Esperança do Sul, Fazenda Itaquerê, left margin of Jacaré-Guaçu river, under rotten plank, very humid place, 20.VI.1965, K. Lenko col., 2♂; Funil, 28.V.1902, O. Dreher col., Departamento de Zoologia São Paulo, 2♀, 2♂; Franca, XI.1902, O. Dreher col., Departamento de Zoologia São Paulo, 1♀; Indiana, IV.1944, Dirings, 1♀; same locality, 02.III.1935, 1♀, 1♂; same locality, II.1935, Departamento de Zoologia São Paulo, 1♂; same locality, II.1934, Departamento de Zoologia São Paulo, 1♂; same locality, XI.1935, 2♀; Onda Verde, Fazenda São João, I.1946, F. Lane col., 1♀, 2♂; Pirassununga, Emas, without date, Schubart col., 1♀; São Roque, 1961, H. Lane col., Departamento de Zoologia São Paulo, 2♀; without locality and date, 1♀; Ribeirão Preto, Tamanduá, 15.XI.1954, Barreto col., 1♂; same locality, 1896, Departamento de Zoologia São Paulo, 1♀; same locality, X.1954, Barreto col., 1♀. Paraná: Curitiba, 1938, Departamento de Zoologia São Paulo, 1♂; Porto Tibiriçá, Rio Paraná, IX.1938, 3♂; same locality, IX.1940, 1♀. Santa Catarina: Anita Garibaldi, VII.1929, Dirings col., 3♀, 1♂; same locality, VI.1929, Dirings, 4♂; Nova Teutônia (currently Seara), II.1966, F. Plaumann col., 2♀, 1♂; Rio Grande do Sul: Cruz Alta, without date, E. Garbe col., Departamnto de Zoologia São Paulo, 1♂; Porto Alegre, II.1929, Dirings, 1♂. PERU – Inca: Chanchamayo, 1200m, XII.1948, Schunke col., 1♀. ARGENTINA, Salta: San Martin, Pocitos, I.1960, A. Martinez leg., 5♀, 1♂; without locality, III. 1958, Martinez Pereira col., 3♀, 4♂. Misiones: Leandro N. Alem, XII.1961, A. Martinez leg., 1♀; Puerto Esperanza, II.1961, A. Martinez leg., 1♀, 1♂; Dos de Mayo, 350m, X.1971, 2♀, 3♂. BOLIVIA, Santa Cruz: Chapare, 400m, Dirings, 1♀, 1♂; Parapeti, X.1960, A. Martinez leg., 1♀; Provincia de Ichilo, Buenavista, II.1950, A. Martinez leg., 1♀, 3♂; Santa Cruz de La Sierra, 08.III.1954, Bruira col., 1♀. PARAGUAY, Presidente Hayes: Puerto Elsa [Puerto Elisa], XI.1936, 1♂; without locality, XII.1950, Dirings, 1♀, 1♂. All material is from MZSP.

##### Key for the immatures of Galeritini with known pupa

**Table:** 

1	Third instar larva: antenna longer than head width; head length more than four times longer than the nasale. Pupa: lateral projections without setae at the apex	*Galerita carbonaria*
–	Third instar larva: antenna shorter than head width; head length less than three times longer than the nasale. Pupa: lateral projections with a pair of short setae at the apex	*Trichognathus marginipennis*

##### Relationships

In his monograph, Reichardt (1967) included a pre-cladistic reconstructed phylogeny in which *Trichognathus* was proposed as the sister group of the Old World Tropical *Eunostus* Castelnau, 1835. According to Ball (1985) and Hunting (2008), however, *Trichognathus* is postulated to be sister group of *Galerita sensu lato* (*Galerita* *sensu stricto* + *Progaleritina*), together forming the sister group of *Ancystroglossus* Chaudoir, 1862. Detailed studies on immature characters may contribute to the resolution of phylogenetic relationships within the group.

##### Natural history notes

Adults and larvae of *Trichognathus* are fast running predators and go in search of their prey at night usually on sandy beaches along rivers and small streams in grassy areas, as well as in gallery forests (Arndt and Drechsel 1998). Adults and larvae are seen hunting together. No one knows for sure what kind of prey they eat, but based on detailed study of mouthparts, Ball (1985) postulated that the very specialized mouthparts play an important role in the capture and manipulation of food.

**Figure 7.**
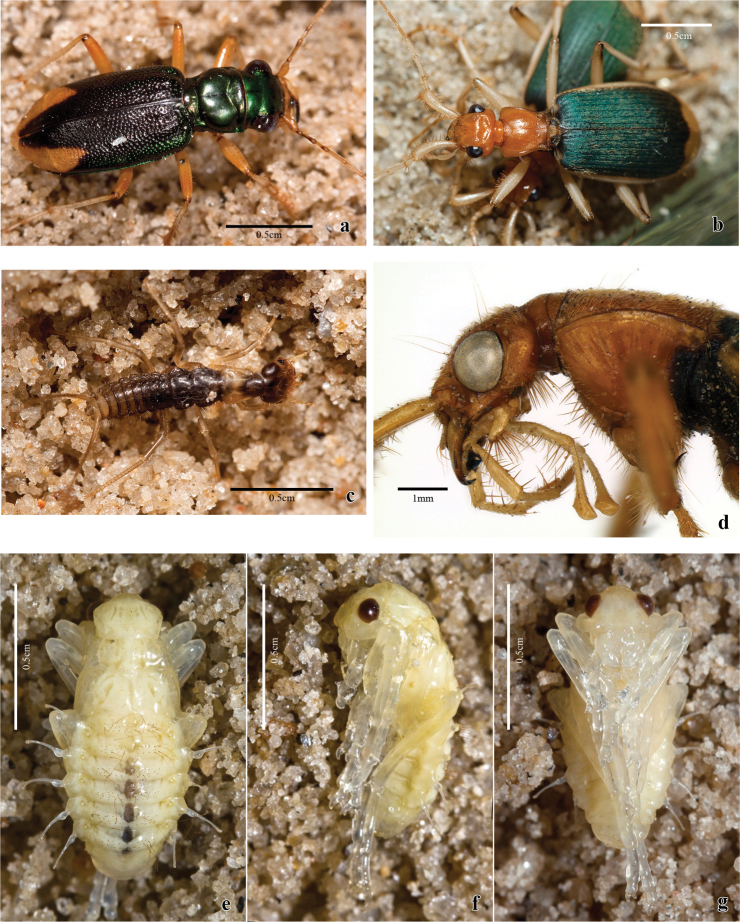
**a** *Tetracha* sp. **b,c,d,e** *Trichognathus marginipennis* **a** *Tetracha* sp. collected with *Trichognathus marginipennis* **b** dorsal view of adults **c** living larva in dorsal view **d** head and prothorax of the adult in lateral view; pupa **e** dorsal view **f** lateral view **g** ventral view.

Larvae and adults were found together on sandy substrate along a river margin, where they were active from sunset until about two hours later; after this period I found no more carabid larvae and adults. Sharing the same environment, some carabid adults of the genus *Tetracha* Westwood (Fig. 7a) were observed hunting. Possibly the same fact was observed by Arndt and Drechsel (1998) that indicated a frequent association with individuals of the carabid *Megacephala* Latreille, but now the genus *Megacephala* is divided into seven genera and *Tetracha* includes only Neotropical species. The group currently accepted as *Megacephala* occurs only in sub-Saharan region (Naviaux 2007).

Gregarious behavior was observed (Fig. 7b), as well as cannibalism among adults of *Trichognathus marginipennis* in the laboratory. During the pre-pupal stage (about two days before the pupal stage), the larva, usually agile, stayed motionless, writhing only when prodded; the pupal period lasted about one week. After death, the darkening of the head and pronotum of adults was also noticed.

##### Comparisons

**Figure 8.**
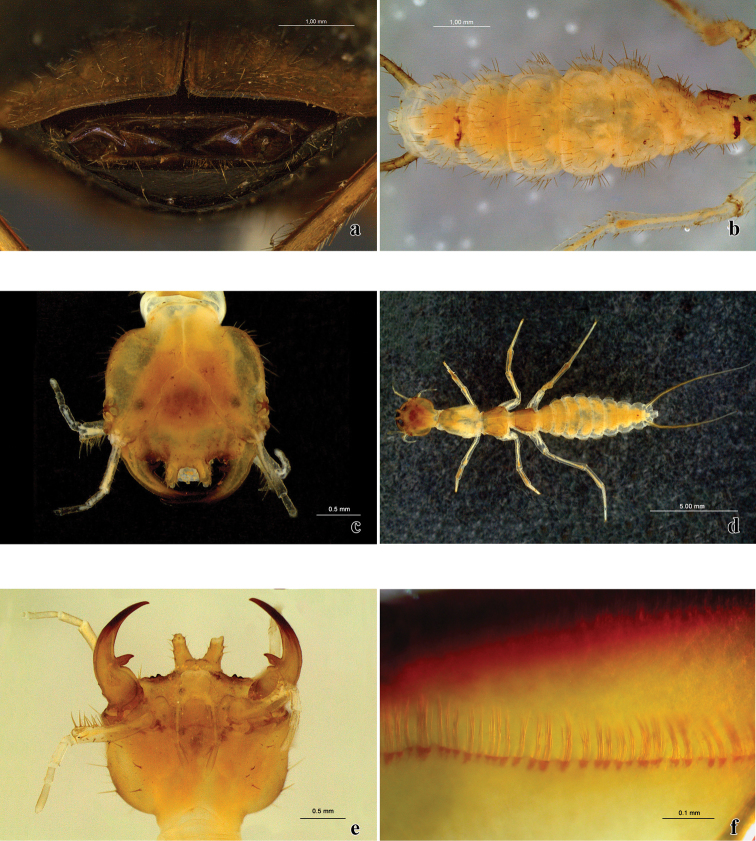
*Trichognathus marginipennis*: **a** gonocoxites from the outside **b** abdominal segments of the larva in ventral view **c** head of the larva in dorsal view **d** larva in dorsal view **e** head of the larva in ventral view **f** detail of the mandible filtering apparatus.

The description of the third instar larva of *Trichognathus marginipennis* made by Arndt and Drechsel (1998) is quite complete and a full redescription is not necessary. However, unlike the original description of the larva, I observed that the abdominal sternites are distinct (Fig. 8b) and not fused as previously described. Furthermore, four bristles were found on the right side of the ventral surface of the head (Figure 8e) in one specimen; usually, three are present on each side.

Comparing the pupae of *Trichognathus marginipennis* with those of *Galerita carbonaria*, showed that they are quite similar, with the following differences: each lateral abdominal extension has a pair of setae at the apex and all thoracic segments with some short setae in *Trichognathus marginipennis*, whereas in *Galerita carbonaria* there are no such setae. Moreover, the eyes of the pupa of *Trichognathus marginipennis* are apparently a bit larger.

As noted by Reichardt (1964), there are no consistent geographical patterns among the described morphs noted above. The genitalia of some females were dissected and the fairly long spermathecal gland, considered a remarkable character by Hunting (2008), was not observed, appearing only slightly elongated (Figure 6c). The sexual dimorphism of dilated tarsi was observed more pronounced in some males than in others.

**Figure 9.**
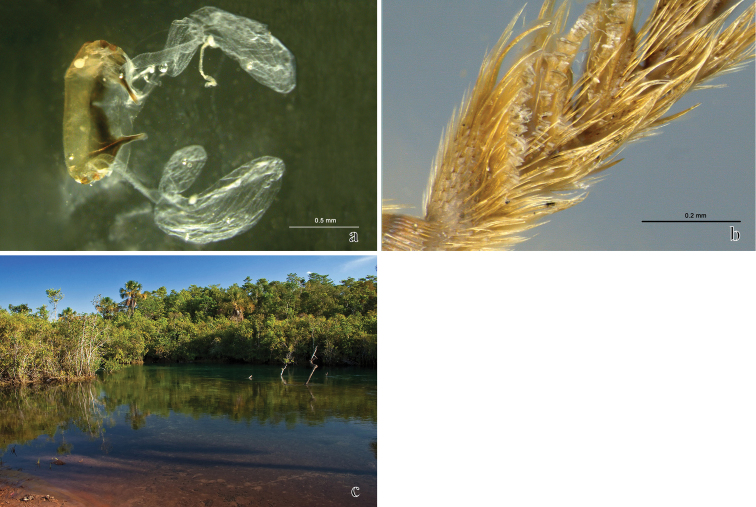
*Trichognathus marginipennis* (adult): **a** sternite VIII with pygidial glands **b** male fore tarsomeres 1–4 in ventral view **c** Rio Verde view from Fazenda Soledad.

## Supplementary Material

XML Treatment for Trichognathus marginipennis
